# Working Under the Gun: A Theoretical Analysis of Stressors Associated With the Re-negotiation of Norms and Control of Work Tasks During COVID-19

**DOI:** 10.3389/fpsyg.2021.577769

**Published:** 2021-08-27

**Authors:** Leo Kant, Elisabeth Norman

**Affiliations:** ^1^AFF at the Norwegian School of Economics, Bergen, Norway; ^2^Faculty of Psychology, University of Bergen, Bergen, Norway

**Keywords:** benign violations, job stress, crisis management, norms, dual thresholds, counterproductive work behavior, professionalization, leadership

## Abstract

The COVID-19 pandemic has led many of the world's nations to impose numerous preventive and mitigative measures to increase social distance, including various forms of home isolation and quarantine. A central premise for the current paper is that the COVID-19 situation is likely to constitute a massive re-negotiation of social and organizational norms, which may lead to psychological distress at the individual, family and interpersonal level. Virtually overnight, people have to re-define what is expected and deemed appropriate by a given group member in a certain social setting. This goes for all kinds of general social interaction, such as societal, even multinational medical demands on social distancing. Simultaneously it also goes for a sudden, gargantuan re-division of labor in a complex global system. We provide a theoretical analysis of the potential consequences of re-negotiation of norms from the perspective of four sets of psychological theory: Theory of professions; organizational strategic crisis responses; the job-demands-resources model; and theories addressing the interplay between norm violations and psychological distance. From these theories we derive three suggestions that the discussion centers around: (1) The COVID-19 situation leads to a massive re-negotiation of norms related to work, (2) The COVID-19 situation diffuses the demarcation between the various professional arenas and the private sphere, and this diffusion enhances the stress associated with norm conflict, and (3) Norm conflicts are enhanced by digitalization. Our discussion centers on potential stressors associated with the renegotiation of norms, and also includes a few suggestions for practice. For each theoretical suggestion, we give examples of how the suggestion may manifest itself with respect to (a) the work task, (b) the individual's relationship to their leader and/or organization, and (c) interpersonal relationships. We finally point to some theoretical and applied implications.

## Introduction

The COVID-19 pandemic has led many of the world's nations to impose numerous preventive and mitigative measures to increase physical distance between people, including various forms of home isolation and quarantine. By March 30th 2020 it was estimated that such measures affected 43% of the planet's population across 78 nations (Norwegian News Agency, 2020). We argue that such measures may cause a number of social norm negotiations, re-negotiations, and conflicts (e.g., Asch, 1951; Cialdini and Trost, 1998), and we address conflicts that may arise when the majority of the workforce suddenly is instructed to work from home during a situation like the COVID-19 pandemic.

Our main argument is that the current situation is likely to constitute a massive re-negotiation of social, organizational, and behavioral *norms*, which may lead to psychological distress at the individual and interpersonal level (cf. Thompson and Hart, 2006). The interpersonal level includes family, work, and other social interaction. Virtually overnight, people have to re-define what is expected and deemed appropriate by a given group member in a certain social setting. This goes for all kinds of general social interaction, such as societal, even multinational medical demands on social distancing. Simultaneously it also goes for a sudden, gargantuan re-division of labor in a complex global system. What happens to *the work task* stands front and center in understanding the re-division of labor (cf. Abbott, 1988).

In this paper, we address the potential consequences of this re-negotiation for workers in organizations. We are above all interested in the “social construction and sensemaking” close to the individual, which happens at the *nano level* (Thompson and Hart, 2006, p. 231). In other words our focus lies with *behavioral norms* created at the nano level, not the moral principles created at the macro level (Thompson and Hart, 2006).

## Research Question and Aim

We address the following research question: What type of role/work stressors is the COVID-19 situation most likely to entail for those who have had to work from home as the result of societal and organizational responses to the pandemic? We provide tentative answers to this question, formulated as a set of suggestions, by combining four sets of psychological theory. In addition, we formulate two more specific propositions that can be tested empirically and that can be used to inform practice.

Our primary aim is to combine existing psychological theory from different areas of psychology in order to provide a theory-based discussion of the types of role/work stressors that are likely to be associated with the COVID-19 situation. A secondary aim is to suggest some theoretical links between the four theoretical approaches, with a particular focus on how the COVID-19 situation exemplifies the general applicability of the Benign Violation Theory to situations of norm violations (cf. Kant and Norman, 2019).

Our discussion centers around the following theoretical suggestions: (1) The COVID-19 situation leads to a massive re-negotiation of norms related to work, (2) The COVID-19 situation diffuses the demarcation between the various professional arenas and the private sphere, and this diffusion enhances the stress associated with norm conflict, and (3) Norm conflicts are enhanced by digitalization. Our discussion centers on potential stressors associated with the renegotiation of norms, and also includes a few suggestions for practice.

Four features of the COVID-19 crisis are central for our further analysis. First, it is *global*. Second, its nature and the mitigation efforts lead to direct authority-ordered imperative *changes for many forms of social interaction, including working life*, as well as indirect changes through organizational strategic crisis responses. Third, *the pandemic is but one of several* associated crisis trajectories (e.g., Oxfam, 2020). Fourth, the pandemic alone appears to have *a long duration* (Kissler et al., 2020; Moore et al., 2020). Combined with associated crisis trajectories the duration appears more certain and likely even longer.

## The Scope of Our Paper

COVID-19 affects the entire planet. A large part of the population, if not all, has been or will be affected by some form of quarantine measures. During shelter-in-place and quarantine measures, work has to be adjusted or radically changed. Such changes demand social renegotiation related to work. The changes related to work have some commonalities, and some differences. Commonalities may range from hygiene measures to changed work tasks, new job demands, and ambiguous norms. Differences are for the purposes of this paper above all whether it is possible to work from a distance or not.

This paper specifically addresses stressors for those who are currently employed, but who have had to *work from a distance* during the pandemic. For whom, amongst other stressors, ambiguity between the private and the work sphere increases, as does ambiguity between their work sphere and that of household members.

Thus, our paper does not address work life for essential workers who still have a job and have to do it physically present. For instance, health care workers, store clerks, transport operatives, garbage disposal workers, farmers, and many others. We do also not discuss stressors specific to *workers without a choice*, e.g., many in the “gig economy” who are forced out regardless of risks, or even while sick. If they do not work they cannot feed themselves or their dependents. The latter group may also include a larger proportion of those who quickly face unemployment.

However, in spite of the somewhat narrow focus, the implications of our work are potentially broad. This is due to the fact that investigating the chosen population promises more than knowledge for practical solutions during the COVID-19 crisis. It also holds promise for the future, far beyond a couple of 100 million privileged workers today. Understanding social renegotiation of distance work, is for instance relevant: (a) in light of a general digitalization trend; (b) as a seemingly necessary strategy during any future pandemic; and (c) as a potential strategy against other dilemmas for a rapidly increasing world population, including global warming, large conflicts, and commuting logistics in urban mega-cities.

The aim is to put forward some theory-based suggestions for how the pandemic is likely to influence work life for the individual. Overall, this attempt exemplifies how it is possible to analyze complex situations by combining different sets of psychological theory. We do not aim to present a new theoretical framework to integrate different theories, but rather to show how different theories applied to a larger problem together can constitute the basis for empirically testable suggestions. Since the paper was written at the very start of the pandemic, it does not incorporate empirical evidence of what is so far known about the actual consequences of the pandemic. However, empirical verification or falsification of our suggestions can and most likely will be dealt with by a follow-up paper.

## The Search for Relevant Theory

Our motivation for writing this paper was a deep sigh shared with millions around the globe. In line with everyone else who had to change aspects of their work life from one day to the next, we experienced stress, norm conflicts, and mismatched experiences in relation to our own work obligations and in relationship to work life of other family members. This made us curious as to how we can use psychological theory to understand the stressors and conflicts that characterize work life during a pandemic, and to make predictions about what types of norm conflicts are likely to arise at different levels of analysis.

The selection of relevant theories was based on a combination of different criteria. First, we wanted to include theories that address *norms* for appropriate vs. inappropriate behavior. Second, we searched for theories that were *universal* rather than COVID-19 specific, yet sensitive for crisis conditions. Third, theories had to be applicable to *working life*—in the sense that they describe processes that are regular responsibilities of leaders and managers, and address outcomes of stress and conflict that are likely and measurable among workers. Fourth, they should describe how *dilemmas* may be addressed through choices or negotiations. Our aim was to address a set of theories that would jointly cover a range of levels of analysis (i.e., individuals/dyads/groups/organizations/societies), and that would be complementary to each other and preferably be possible to link to one another.

## Theoretical Framework

Based on the aforementioned criteria, we selected four theoretical frameworks which constitute the starting point for our analysis. First, we consider *theory of professions* (e.g., Abbott, 1988), which describes general mechanisms of control of work tasks, applicable in dramatic as well as less dramatic times. Mechanisms can be applied from macro to micro levels of analysis; from the societal to the workplace; between as well as within organizations. Secondly, we consider *strategic organizational responses to crisis* (Wenzel et al., 2020), which in turn may strongly moderate contextual conditions for nano level norms and control of work tasks. Thirdly, we consider the *job demands and resources* model, the JD-R (Bakker and Demerouti, 2007) which can aid understanding how re-negotiation of norms and control of work tasks, as well as the organization's response to crisis may manifest in terms of objective and perceived job demands and resources at the individual nano level. Theoretically and empirically, job demands and resources are typically investigated at the workplace and affecting individuals or small groups. Fourthly, we consider models addressing *psychological distance and behavioral norms*, which can help understand causes and effects of perceived norm violations, not the least at the dyadic and individual level.

### Theory of Professions

Some of the potential conflicts that occur during re-negotiation of norms (e.g., De Dreu, 2010) during COVID-19, may be understood in light of *theory of professions* (e.g., Abbott, 1988). This ecological and systemic theory ranges from lower levels of analysis such as a workplace to societal levels.

A profession can be defined as “any occupation that competes for a work via (...) cultural activity” (Abbott, 2010, p. 176). Professionalization theory has *control over the professional task* as a pivotal point. For example, the medical profession has the exclusive control over sticking knives into people—a strong jurisdiction supported by societal level laws, public opinion, and by procedures and division of labor at the workplace arena. As pointed out by Scott (2008), occupational groups also compete about control over advanced technologies and new kinds of knowledge. Professionalization theory outlines a systemic and perpetual contest over such control. Clearly the COVID-19 situation leads to issues concerning control of professional tasks, advanced technologies and new kinds of knowledge.

Professionalization theory is well-suited to explain how the system of professions is influenced by various crises, as well as political or technological changes. Thus, the processes may work over time, or in intense bursts.

The mechanisms may work on a societal level, on a legal arena, or on a workplace arena. Furthermore, the mechanisms are the same *between* professions and *within* professions. Thus, domino effects may occur on several levels, and between and within entities.

Various external shocks, such as technological or demographical events, may reshape the conditions of the perpetual conflict in which professions normally exist (Abbott, 2010, p. 176). The COVID-19 situation clearly involves such “shocks.” The scale, the number of simultaneous and consecutive shocks, and speed associated with COVID-19 are notable. Severe shocks force different types of organizations to choose strategic crisis responses (see the section “Strategic responses to organizational crisis”).

An important point is that legitimate control of professional jurisdictions (Abbott, 1988) requires *legitimacy—*“the belief that authorities, institutions, and social arrangements are appropriate, proper, and just” (cf. Tyler, 2006, p. 376). Legitimate professional control may connote a jurisdiction or area of authority which is defined by law, but also by perceptions in the public eye, or by *psychological contracts* (cf. Thompson and Hart, 2006) at the workplace arena. How people think about such social arrangements greatly influence their willingness to defer to them (Tyler, 2006).

An individual's behavior may be guided by personal conviction. However, notions of professional theory, legitimacy, and psychological contracts clearly point to the power of shared thinking on effective behavioral norms. In the case of stretching, breaching or fully breaking existing psychological contracts, we need to understand how perceived legitimacy and according deference to contracts may begin to waver.

To sum up, changes relating to control of work tasks may relate to who controls the work tasks; who or what has oversight, surveillance, and sanctioning rights (Dzieza, 2020); the relative importance of different work tasks; the status associated with various roles/tasks; the boundaries between professional and private tasks and responsibilities. Changes may also shift the balance of tasks performed in physical proximity vs. distally/digitally. We now turn to each of these shifts.

### Strategic Responses to Organizational Crisis

An organizational crisis occurs when the normal organization, resources, points of reference and sense of meaning are no longer sufficient (cf. Pearson et al., 2007; Narotzky and Besnier, 2014). This would be the case for many organizations during COVID-19. In times of crisis the regular division of labor is not enough, especially when the process of crisis escalation is vague and prolonged (Jacques et al., 2007; Buchanan and Denyer, 2013). Management may thus be uncertain about appropriately mobilizing crisis responses, thus displaying passive or laissez-faire leadership (Lewin et al., 1939; Skogstad et al., 2007). The daunting task of controlling the uncontrollable during crises, can even lead to destructive passivity.

In a recent review, Wenzel et al. (2020) present an overview of papers that address ways in which organizations can respond to crises. Wenzel et al. identify four broad categories of organizational strategic responses to crises. These are *retrenchment, persevering, innovating*, and *exit* (Wenzel et al., 2020).

*Retrenchment* involves reducing costs, assets, products, or overhead (Pearce and Robbins, 1994, p. 614, in Wenzel et al., p. 9). This causes the organization's business activities to narrow. A potential result is often a net loss for the organization, and may be detrimental in the long run. A different strategy is *persevering*, where attempts are made to sustain the organization's activity. It can be seen as a “status quo” strategy. In the initial stages of a crisis, persevering may be a rational strategy for various reasons, including the need for more information about the estimated nature and duration of the crisis. However, as pointed out by Wenzel et al. (2020, p. 10), this strategy only works in the medium run, with “slack resources” available. Passivity could be seen as a variety of persevering. *Innovating* involves a change, broadening, or renewal of the organization's scope of business activities. Wenzel et al. (2020) argue that innovating is likely to be beneficial in the long run, and sometimes key to survival of the organization. *Exit* means that the organization discontinues with its activities. Whether an organization chooses this strategy depends on a number of factors, including the nature of the crisis and strategies at earlier stages of the crisis. Sometimes, exit is unavoidable. At other times it may be a deliberate strategy.

With a scope as in the current pandemic, all types of organizational responses, even initial persevering, may eventually lead to great change. We propose that these organizational responses constitute central vessels bringing the change into the work arena. In particular, vessels for change concerning the control of work tasks, and the demands and resources associated with work tasks.

### The Job-Demands-Resources Model

Thirdly, closer to the individual, the *Job-Demands-Resources model* (JD-R; Bakker and Demerouti, 2007), sheds further light on processes concerning work tasks: how changes in demands and resources influence what happens from an organizational level of analysis down to the individual. A sense of control is part of the backdrop to the JD-R model and its resources, for instance as manifested by the name of the earlier *demand-control model* (Karasek, 1979) which the JD-R extended.

The JD-R model has been central for understanding the interplay between two categories of factors that determine the level of stress in a given job situation. Both categories of factors can refer to physical, psychological, social, or organizational aspects of the job. On the one hand, *job demands* refer to job aspects that come at a cognitive and/or emotional cost and may lead to stress, because they require sustained effort or skills. *Job resources* refer to those job aspects that help the person to achieve their work goals, reduce job demands, and/or stimulate growth, learning, and development. Job resources can counter the effect of job demands, but are also valued in their own right (Bakker and Demerouti, 2007). Importantly, both sets of factors can operate at different levels of analysis, including at the work task, leadership/organizational, and interpersonal level. An important assumption of the JD-R model is that job strain and job motivation are influenced by two different psychological processes. Thus, it can be seen as a dual process model (Bakker and Demerouti, 2007). Job demands and resources also interact. For instance, according to the “buffer hypothesis,” job resources can modify the negative effect of job demands on perceived stress (Bakker and Demerouti, 2007).

The JD-R model also acknowledges that *personal resources* (Bakker and Demerouti, 2007) can mediate the relationship between job resources and job engagement, but not buffer job demands on their own (Xanthopoulou et al., 2009). This is relevant during the COVID-19 pandemic, with workers exposed to both increased social demands and reduced job resources.

Bakker and Demerouti (2007) point out that job stress and motivation not only predict job demands and resources, but can also be outcomes of the two. For instance (poor) worker behavior may lead to increased demands and reduced resources over time. Also, a dark perception of demands and resources may lead to a deteriorating work climate and objective worsening of demand-resource combinations. Such reversed causation requires attention to other causes of job stress and motivation. We suggest that the fourth theoretical framework below is useful for this end.

### Models Addressing Psychological Distance and Behavioral Norms

When norms are re-negotiated, this implies changes to what is perceived as acceptable vs. unacceptable, both at the nano level, interpersonal level, and societal level. When psychological contracts are stretched in a manner that violates one's expectations, this violation may be perceived as either benign/legitimate/acceptable or malignant/illegitimate/unacceptable.

*The Benign Violation Theory* (henceforth BVT; McGraw and Warren, 2010; McGraw et al., 2014) explains how emotional responses to norm violations depend on whether the violation is regarded as benign or malignant. This is originally a theory of humor, but can be applied to other situations in which a norm violation leads to emotional reactions (Kant and Norman, 2019). For something to be regarded as funny, two types of appraisal must be present simultaneously. First, something must violate expectations of how something “ought” to be, e.g., violating a linguistic norm. Second, this violation must be perceived as benign rather than malignant and hurtful. As Kant and Norman (2019) argue, other dual threshold models of social behavior describe a similar process where behavior may violate some expectations or norms, while being acceptable in a sweet spot prior to passing into the unacceptable. For instance, concerning anger in organizations (Geddes and Callister, 2007), leadership, or extra-role behavior at work (e.g., Spector and Fox, 2010).

In this paper, we refer to “mismatched experiences” and/or “mismatched sweet spots” in cases where a norm violation is perceived as benign by one person and malignant by another, resulting in different emotional reactions. The term psychological distance is important for understanding norm violations and mismatched experiences (Kant and Norman, 2019). According to *the construal level theory (CLT)* of psychological distance (Trope and Liberman, 2010), something or someone may feel closer or further away in terms of geographical distance, social distance, temporal distance, and hypotheticality. Something which is further away is represented more generally and abstractly, whereas something which feels closer is represented more concretely.

Telecommuting in general, and the COVID-19 situation in particular may influence psychological distance in all its dimensions. Geographical or physical distance to coworkers and leaders is typically increased, particularly so during the pandemic with mitigation strategies against contagion. Interaction frequency may go down, but also increase through online work environments. Social distance—not the least in terms of power asymmetry—may increase, become more unclear, and conceivably also decrease. Psychological distance to people in the same household may move in an opposite direction. The psychological distance to work tasks may also change.

Of special interest here is *leadership distance*, which can be understood in terms of three dimensions, namely, *leader–follower physical distance, perceived social distance*, and *perceived task interaction frequency* (Antonakis and Atwater, 2002). Leader-follower physical distance refers to the geographical distance between leader and follower. Perceived social distance refers to differences in status, rank, authority, social standing, and power. These are assumed to influence perceived intimacy and social contact. Perceived task interaction frequency refers to how often the leader and follower interact, either face to face or digitally.

Geographical and temporal distance moderates norm violations (McGraw et al., 2014). When something *feels* close to us, it takes a smaller violation for it to be considered malignant. A higher intensity is possible when distance is greater. Sweet spots in leader-follower interactions related to the dimensions of leadership distance have also been substantiated empirically (Vanderstukken et al., 2019; Gouldman and Victoravich, 2020; Hernández et al., 2020).

Third, differences in perceived psychological distance between two parties may cause mismatched experiences of whether a violation is benign or malignant (Kant and Norman, 2019). Also, the relative distance to the norm-violation itself (here the work task or changes in demands or resources) works in the same way. For instance, the psychological distance to other parties or to a norm-violation will likely be experienced differently by a high-power party–such as a leader, an official, or a member of a dominant social group, compared to a low-power party–such as a worker, citizen, or member of a subordinate group. The changes and renegotiations during COVID-19 should therefore be considered in terms of their potential for mismatched experiences.

## Discussion

We now present 3 theoretical suggestions for what type of role/work stressors the COVID-19 situation is most likely to entail for those who have had to work from home as the result of societal and organizational responses to COVID-19. The stressors are related to different factors over which people currently have to re-negotiate. These suggestions are derived from our knowledge of the four sets of theory presented in the previous section of the paper.

**Figure 1:**
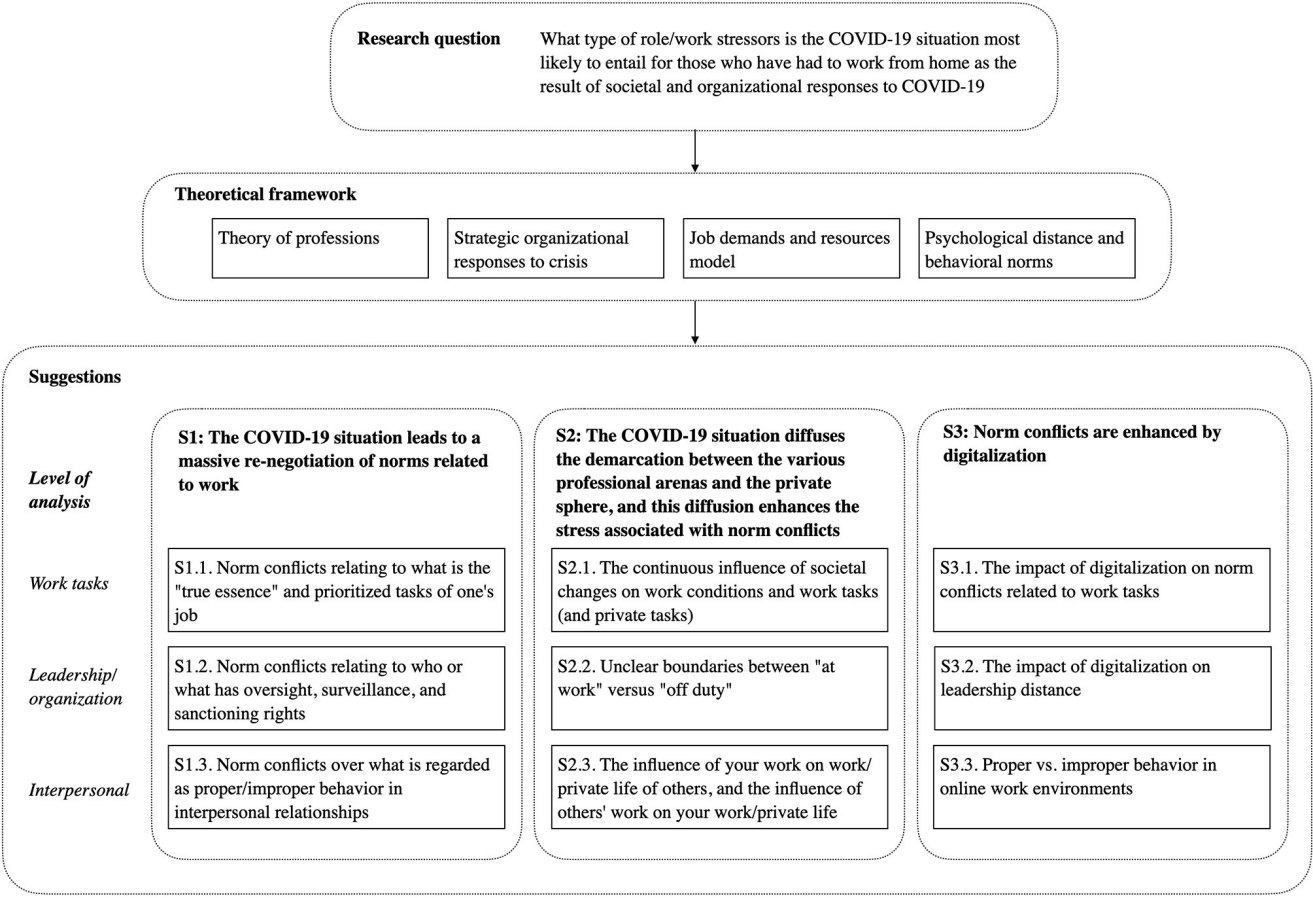
A graphical overview of the logical flow of the paper.

For each suggestion, we first give a general introduction to what the suggestion implies at a general level. We then provide 3 sets of examples, at the level of the *work task, leader/organization*, and *interpersonal relationships*, respectively. We supply a graphical overview of the suggestions in Figure 1.

### Suggestion 1: The COVID-19 Situation Leads to a Massive Re-negotiation of Norms Related to Work

The COVID-19 situation involves multiple, massive changes at the societal, organizational, dyadic, and individual levels. People have to reorient themselves about what is expected, acceptable, etc. We refer to the process of establishing new norms as “re-negotiation of norms.” We will address 3 sets of factors over which people now have to re-negotiate. There may also be other factors.

Using the terminology of the Benign Violation Theory (McGraw and Warren, 2010), norm re-negotiation implies that what yesterday was acceptable behavior, may today be unacceptable. To use the terminology of BVT: The dual thresholds have moved. Different parties may experience different shifts in dual thresholds, the contextual premises, and psychological distance to other parties and to the subject matter. In line with our hypotheses put forward elsewhere (Kant and Norman, 2019) we therefore expect *widespread and frequent experienced norm violations* of both benign and malignant nature during COVID-19. Above all, we expect many *mismatched experiences*, where one party perceives a violation where the other does not.

Moreover, we suggest that *the mere re-negotiation of norms could itself be seen as a stressor*. Re-negotiation is a general stressor in society, and a variable that increases perceived job demands in connection to work (cf. Bakker and Demerouti, 2007). By this we mean that both *uncertainty* (“Is task X important anymore?,” “By which authority am I to do the old X or the new Y?,” “Will I get paid for doing X or Y?”) and *change* (“It seems I can't carry on doing what I normally do.”) might demand more of the individual. The *result* of this re-negotiation (“I have to do more of Y and less of X.” “I seem to have less autonomy doing Y”) could also be demanding.

The violation of norms is likely to be strenuous: cognitively as represented in the previous questions, and by a more than usual need to suppress, enhance or fake emotions (cf. Glasø et al., 2006; Gailliot et al., 2007; Bushman et al., 2014).

Importantly, norm re-negotiation could take place overtly—with people being aware that re-negotiation takes place. Yet, it might also occur covertly—with people being unaware of the re-negotiation process or how their communication might be perceived. Regardless of people's awareness of the process, we expect that most people are tired, confused, and step on each other's toes more than before.

**Figure 2:**
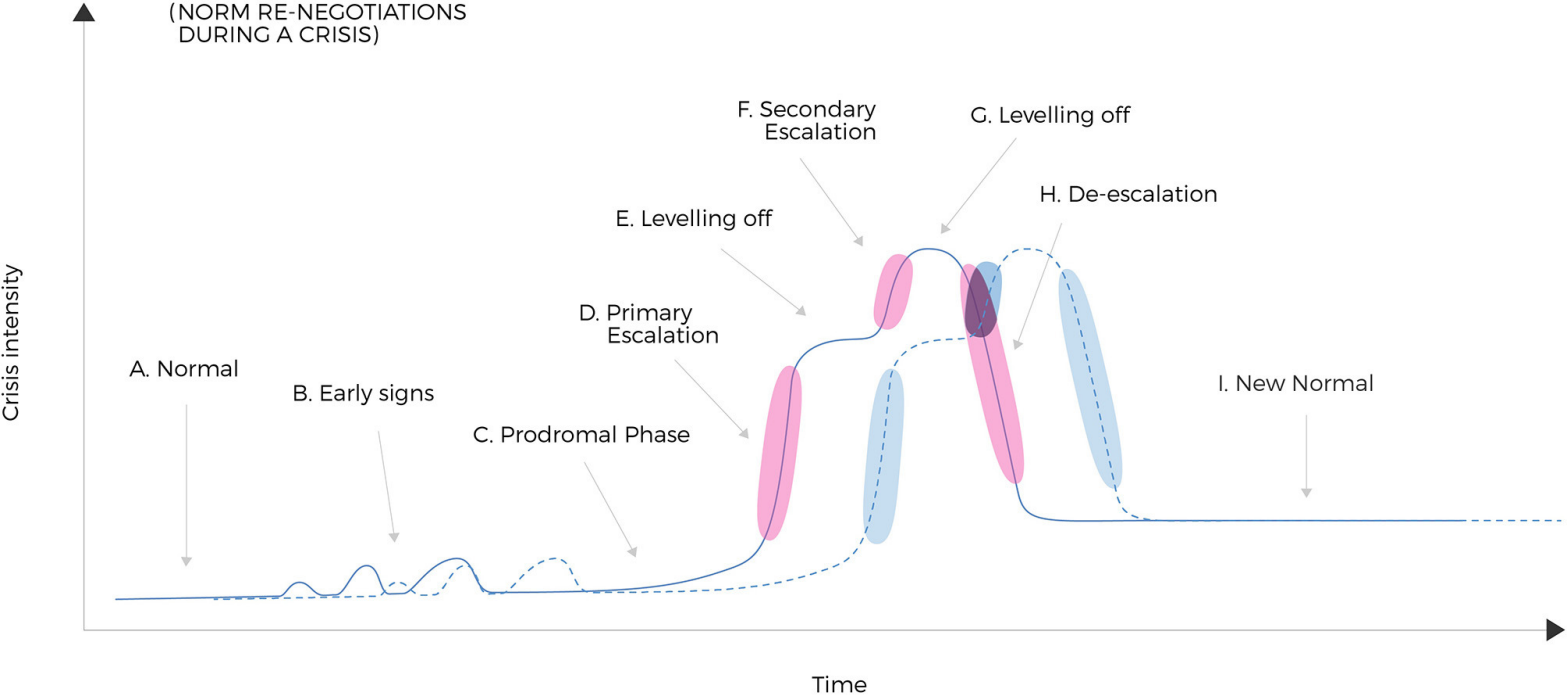
Norm negotiations over time across different stages of a crisis (crisis curve = solid line). The figure gives a graphical overview of how norm negotiations develop as a function of crisis intensity and time. We divide the crisis into the following stages, with reference to literature which the labels either originate from or are inspired by: **(A)** Normal; **(B)** Early signals (Mitroff, 1988); **(C)** Prodromal phase (Fink, 1986); **(D)** Primary escalation (Fink, 1986; Curtin et al., 2005); **(E)** Levelling off (cf. Curtin et al., 2005); **(F)** Secondary escalation (Fink, 1986; Curtin et al., 2005); **(G)** Levelling off (cf. Curtin et al., 2005); **(H)** De-escalation (Fink, 1986; Mitroff, 1988; Faulkner, 2001; Curtin et al., 2005; Jacques et al., 2007); **(I)** The new normal (cf. Pearson and Clair, 1998; Ritchie, 2004). A second crisis curve (dashed line) indicates that a crisis may be accompanied or followed by other crises such as the economic crisis following the medical crisis of COVID-19. Importantly, we have indicated the timing of increased likelihood of re-negotiation of norms. Times of strong re-negotiation is indicated by clouds in phases where there are strong changes in intensity (escalation or de-escalation), i.e., during phases **(D,F,H)**. Note that on two occasions such clouds on both crisis curves coincide in time, which could possibly lead to interactive or additive effects.

We specifically hypothesize that the intensity of social renegotiation is positively correlated with the slope of change in crisis intensity (both increasing and decreasing slopes). We provide a tentative, graphical overview over how we hypothesize the intensity of re-negotiations of norms will vary across different stages of a crisis (see Figure 2). Our overview builds on established research on how a crisis is known to develop (see references in caption to Figure 2). Simply put, we predict re-negotiation to peak while societies close down as well as when they are opening up. During lock-down, particularly with prolonged duration, temporary settlements will calm things down. During a de-escalation phase (e.g. Fink, 1986; Mitroff, 1988) we however hypothesize greater variance in norms and behavior, than during the escalation. Thus, norm conflicts may increase even more during de-escalation.

#### The Level of Work Tasks: Norm Conflicts Relating to What Is the “True Essence” and Prioritized Tasks of One's Job

The first set of factors over which workers have to re-negotiate, concerns the work tasks. Some central questions are: *What is the “true” essence of my job? Which are the prioritized core tasks? What is true in a short term crisis vs. a long term one?*

##### Changes in Nearly all Aspects of Work Tasks

Physically moving one's workplace from the site of the organization to one's home, may require brutal prioritization of certain tasks at the cost of others. First, there might be work tasks that simply cannot be conducted from a distance or digitally, i.e., by telecommuting. Second, the work situation might lack sufficient time and resources to do all the tasks one normally is responsible for. In theoretical terms—a sudden shift in job demands and resources (cf. Bakker and Demerouti, 2007). Possibly related to limitations to technical equipment, or having to distribute one's time between work and taking care of quarantined household members. Third, the organization's crisis response (cf. Wenzel et al., 2020) could cause changed task prioritization.

##### What Is the Essence of My Job?

Especially with organizational retrenchment or innovation crisis responses, workers are forced to consider the “true” essence of their job. Some might have to do the tasks of the lost workforce. Someone in an organization suddenly re-defining its goals and priorities, might have to use their skills differently. With an organization that attempts the perseverance strategy, the initial phase will likely lead to differentiated work-load and prioritization of work tasks within the organization. Some, like crisis managers and IT-technicians, may have an intense workload with both old and new work tasks. Others may have very little to do, and ambiguous conditions for taking initiative, prioritizing, and even being visible (cf. Elsbach et al., 2010).

##### Unclear Expectations and Lack of Choice

One thing is what happens to explicitly defined tasks, i.e., tasks that are clearly described and with clear expectations. Another is what happens to tasks that are less clearly defined, and to possible flexible space in between tasks. With a crisis and some response strategy declared, the first casualty in professional control and autonomy (cf. Abbott, 1988) is likely the flexible space in between work tasks, much like the short seconds in between tasks at the conveyor-belt (cf. Dzieza, 2020). Less clearly defined tasks may still be expected to be performed, but without time or other resources. New tasks may similarly have been added, without time, resources, or even pay. The teacher may have to do janitorial and cleaning tasks in the partially opened school, at the same time as having to be a purchaser of IT gear at home, performing janitorial tasks at home, and providing tech-support for the home-schooled children. This in addition to revising curriculum and pedagogical methods to suit the new constraints either in a partially opened school, or in an entirely virtual format.

Meeting the expectations heavily dictated by the organization's chosen strategic crisis response (cf. Wenzel et al., 2020) may thus be a conflicted battle between nominal core tasks on one hand, and implicit, new, and other people's tasks on the other. This, while interstitial “empty” time slots likely have disappeared.

What if a worker tries to say no? Strongly attempting to enforce previous “peacetime” division of labor and work tasks may be perceived as a malignant violation (cf. Kant and Norman, 2019) by leaders and colleagues, even by family members in a shelter-in-place situation.

Should workers display *counterproductive work behavior* (CWB, Spector and Fox, 2010), then increased job demands and reduced job resources could follow, as specified in the JD-R model (Bakker and Demerouti, 2007). Their *negative perception* of available JD-R may also lead to a deterioration. A mismatched perception as described by Kant and Norman (2019) with psychological contracts violated (Thompson and Hart, 2006) may thus lead to both of those mechanisms for reversed causation in the JD-R (cf. Bakker and Demerouti, 2007). If violations in the eyes of workers are experienced as *illegitimate* (cf. Tyler, 2006) worker behavior or perception may be even worse.

##### Re-negotiation as an Opportunity

For some, renegotiation is an *opportunity*. Turmoil provides opportunities to grab new jurisdictions for entire professions (Abbott, 1988), and new market shares and innovations for organizations (Wenzel et al., 2020). Also, at the workplace level, individuals may completely fulfill every expectation, thus demonstrating that they are essential workers and at least should be prioritized to continue having a job. They may even display desirable *extra-role behavior* such as *organizational citizenship behavior* or OCB (Spector and Fox, 2010) meriting for new responsibilities or even promotion. Incidentally, we consider the latter to be a potential for the reversed causality in JD-R. Yet, the opportunities for the worker may lead to violations from the employer to be experienced as benign, and thus help them cling on to the edges of the best JD-R quadrant (average strain and high motivation) rather than dropping into the worst as perhaps otherwise expected (cf. Figure 7 in Bakker and Demerouti, 2007, p. 320). Thus, the same basic mechanism of shifting norms—moving goal posts—may explain why leaders suddenly may perceive the worker's helpful OCB as harmful CWB, and why workers suddenly may perceive constructive leader behavior as destructive (cf. Einarsen et al., 2007). This mechanism may be a useful contribution in answering the question Spector and Fox (2010) pose about the relationship between OCB and CWB.

The first step for taking advantage of the opportunities is probably hitting the sweet spot of one's leader, and perhaps colleagues. Can it be an acceptable violation in the short term? Then perhaps it can be a winning gamble in the long term. To a degree this poses an individual level parallel to the organizational strategic crisis responses. Yet, many are not at liberty to choose at all. They may instead experience extreme versions of professional or workplace lock-in (Aronsson et al., 2000).

#### The Level of Leadership/Organization: Norm Conflicts Relating to Who or What Has Oversight, Surveillance, and Sanctioning Rights

The second set of factors over which re-negotiation now takes place, concerns power distribution: During the COVID-19 pandemic, who or what has oversight, surveillance, and sanctioning rights? For the focus of the current paper, these questions mainly concern the individual's work tasks. However, an understanding of oversight, surveillance, and sanctioning rights of an individual's work tasks also requires power distributions at a societal level.

##### Above and Beyond the Call of Duty

At a workplace arena, norm conflicts get close to the actual work tasks and the individuals performing them. Irrespective of the organization's strategic crisis response (cf. Wenzel et al., 2020), there will likely be discussions around demands beyond what workers are contractually obliged to do, to ensure the survival of the organization or to help clients. Such requests “above and beyond the call of duty” tap into the realm of extra-role behaviors such as OCB and CWB (Spector and Fox, 2010). Such requests are vague in the sense of being open ended, lacking contractual descriptions, and not waiving the worker from doing all the regular tasks. Especially workers who experience threats to their employment, may attempt to meet both the nominal and the vague OCB expectations. In terms of the JDR model this constitutes an increase in demands, reduction of resources and control (cf. Bakker and Demerouti, 2007), which in itself approaches unhealthy work conditions (Karasek, 1979). If combined with fear of losing one's job, the *lock-in effects* come into play, further straining the worker (Aronsson et al., 2000). Who actually asks for the OCB, judges and sanctions whether it has been fullfilled, is likely to be unclear to the individual worker. If it is equally unclear which resources are available, and where their responsibility begins and ends in relation to other work tasks of other workers—the norm conflicts have truly landed in the individual.

##### Exploiting Norm Conflicts

Norm conflicts can also be exploited, both by individuals and groups, including professions. When previous jurisdictional settlements are upended opportunities may reveal themselves (Abbott, 1988). Individuals or groups may be able to get defined as an essential worker, or expand their jurisdiction into more attractive and lucrative tasks. Organizations may be able to grab market shares or venture into new markets. We must remember that (a) norm conflicts carry the seed of both threats and opportunities, and (b) they exist in a system, where movement in one part often influences other parts. This realization is helpful not only in understanding resistance or absence of it toward change (cf. Oreg, 2006). It is also helpful in practice to avoid these processes stealing unnecessary energy.

#### The Interpersonal Level: Norm Conflicts Over What Is Regarded as Proper/Improper Behavior in Interpersonal Relationships

In a situation where norms are negotiated or re-negotiated, there is a potential that the perception of norms and norm violations differ between individuals (Kant and Norman, 2019). Thus, to understand norm re-negotiation, we need to also turn to the interpersonal level.

##### Norm Violations: the Line Between the Acceptable and the Unacceptable

Everyday social interaction at work may be tricky. For instance, while one person might find it acceptable to start the working day an hour later than usual during the lockdown, another might find the same behavior unacceptable. Moreover, a norm violation seen by one person as benign, could be perceived as malignant by another. Using the same example, even if two people agree that the COVID-19 situation should not influence when the working day starts, one person might find it funny that another person gets up an hour late, while another could find it unacceptable and annoying. In terms of the BVT (McGraw and Warren, 2010,), the “sweet spot” of different people is mismatched. The risk of this could be increased for a number of reasons during COVID-19. Multiple norms are re-negotiated, and for many people in telecommuting conditions, re-negotiation also takes place largely without face-to-face communication. Accordingly, it becomes more difficult to perceive each other's subtle social signals, including those that communicate what is acceptable and unacceptable. In essence, the limited cues known from virtual teams and telecommuting gets paired with low skills in virtual social interaction (cf. Arvedsen and Hassert, 2020), and paired with an entire norm-system in turmoil.

Re-negotiation of norms in combination with less face-to-face interaction increases the risk of norm violations, which has a number of potential implications. One is that humor becomes a riskier activity. When moment-to moment social feedback is lacking, it also becomes more difficult to judge what is morally/ethically correct. Another is that leader–follower interaction and leadership pertaining to interpersonal behavior becomes more difficult.

##### Interpersonal Difficulties in Crisis Organizations

Three phenomena can illustrate how organization or group level norm conflicts may cause difficulties at the interpersonal level. If awareness, leadership, and crisis management training are lacking, then navigating the new developing norms is likely to be tricky. Knowing basic dance steps is simply advantageous. If no one knows a single step—someone is bound to get stepped on.

First, leadership in crisis may become *heterarchical* as opposed to hierarchical (Nesse, 2017), with multiple formal and informal leaders assuming and giving responsibilities away. In a trained organization this may work well (Nesse, 2017). However, the potential vagueness, role ambiguity or outright role conflicts (Wong et al., 2007) may prove highly troublesome in organizations or teams that are unfamiliar with these new roles (cf. Espevik et al., 2006).

Second, *shared mental models* are important cognitive phenomena during crises (Cannon-Bowers et al., 1993), necessary for establishing of clear roles, shared situation awareness and shared mental models about goals and solutions. Carrington et al. (2019) showed that in prolonged crises, many shared mental models about solutions emerged from the ranks or from lower management levels. If higher management and the organization are unable to communicate and listen well, the best solutions may be overlooked, or even considered malignant violations.

Third, organization level thresholds for behavior, e.g., concerning displays of emotion, may change. A crisis rigged organization is different from the everyday organization. The sense of urgency and narrowed scope of objectives may change norms for interpersonal behavior. Empirically, the dual thresholds (cf. Geddes and Callister, 2007; Kant and Norman, 2019) was found to shift for crisis managers' aggressive behavior (Larsson et al., 2001): expressing some anger and other emotions was more tolerated, even expected. Yet, the maximum acceptable expression was reduced. In other words, more welcome to show, but with a stricter upper limit. However, workers may be less welcome to show any aggression—needing to inhibit, to perform emotion work in order to keep their job (cf. Glasø et al., 2006). The norms for displaying other emotions may similarly be shifted in line with dual threshold models (cf. Geddes and Callister, 2007; Kant and Norman, 2019). Displays of joy in a hospital ICU-unit, or jokes at the COVID-19 presidential task force press briefings may have changed norms.

##### Emotional Responses to Perceived Malignant Violations

In addition, follower frustration and their behavior may bring existing norm conflicts to the surface, particularly in the eyes of leaders. If we consider that workers may experience the re-negotiation process, the (limited) level of support from leaders and the organization (cf. Bakker and Demerouti, 2007), the resulting work tasks (see section The Level of Work Tasks: Norm Conflicts Relating to What Is the “True Essence” and Prioritized Tasks of One's Job) and associated new control as *malignant violations* (cf. Kant and Norman, 2019) the perceived strain would likely increase even more. If the worker experiences malignant violations, the most immediate outcome is *emotional responses* (cf. Warren and McGraw and Warren, 2010). Merely *inhibiting emotions* is literally energy draining—each act of inhibition gnaws on one's glucose level (Gailliot et al., 2007), and could result in the display of counterproductive behavior (Bushman et al., 2014). Even under normal circumstances, workers and leaders engage in a lot of *emotion work*—inhibitions and exaggerations of actual emotions (Glasø et al., 2006). It is fair to expect that emotion work gets even more intense when uncertainty is high, when norms are unclear, and when employment is at risk, as during COVID-19. As mentioned in The Level of Work Tasks: Norm Conflicts Relating to What Is the “True Essence” and Prioritized Tasks of One's Job, the strain of inhibition and emotion work, and the emotions themselves may fuel both perception and behavior, which may have a detrimental impact on job demands and resources (cf. Bakker and Demerouti, 2007).

Extra-role behavior may be both desirable and highly undesirable. Perhaps a steep curvilinear effect may be found in high turmoil/crisis conditions. It is conceivable that some OCB—above and beyond the normal call of duty—is expected, but not much, lest you quickly irritate colleagues and leaders by doing anything but the core tasks.

### Suggestion 2: The COVID-19 Situation Diffuses the Demarcation Between the Various Professional Arenas and the Private Sphere, and This Diffusion Enhances the Stress Associated With Norm Conflicts

A premise that might shape the nature and outcome of re-negotiation of norms, is the fact that societal restrictions following COVID-19 has caused different arenas to be “merged” into people's homes. Discussions and negotiations normally belonging to the societal and organizational arenas, now occur in the home arena. For instance, active negotiations with one's boss about the distribution of work tasks, could occur in the bedroom. Board meetings have people working from their living rooms, kitchens, bedrooms, etc.

One implication is that the demarcation between various professional arenas and the private sphere, is diffused. In the following, we exemplify how this diffusion may enhance the stress associated with norm conflicts at the 3 levels of analysis.

Our suggestion is that the technological and demographic changes (or “shocks”) occuring as a direct or indirect result of COVID-19, may reshape the conditions of the perpetual conflict professions normally exist in (Abbott, 2010). They also influence work and private conditions of individuals more and quicker than under normal circumstances.

According to theory of professions the struggle about control of work tasks occurs in 3 arenas: the workplace, the public arena, and the legal arena (Abbott, 1988). In the current pandemic, the private sphere is no longer separate from the classical three arenas: This demarcation becomes more diffuse and permeable. Re-negotiation appears to occur in all three arenas as well as the private sphere, simultaneously or nearly so.

We will mainly discuss re-negotiation of social norms in the work arena for people currently working from home. In addition, we point to how the COVID-19 situation challenges professionalization theory's assumption that changes occur at a higher pace in the work arena, followed by the public and legal arenas. For example, some recent changes in the work arena could be seen as direct consequences of more rapid societal changes.

#### The Level of Work Tasks: The Continuous Influence of Societal Changes on Work Conditions and Work Tasks (and Private Tasks)

The COVID-19 situation is like a rhinoceros in a china shop: some professional tasks are put on hold over night (e.g., restaurants, hairdressers, and travel workers), whereas others have a corresponding increase in demand (e.g., health care nurses, laboratory testing, producers of antibacterial liquid), and new ones previously unclaimed ones have emerged (e.g., new business ideas like 3D-printing of face visors on a 1,000 home computers).

The theory of professions (Abbott, 1988) describes how radical changes in control of professional tasks lead to domino-effects, conflicts and re-negotiations. Accordingly, we would expect all three arenas of jurisdictional contest to be in turmoil during COVID-19. Moreover, the changes in all arenas may occur in different ways and in a different order than normally.

Typically, the legal arena is slow to change, the public arena is more dynamic, and the workplace arena is the most dynamic. Presently the workplace arena not only has moved into your home, it is also in minute-to-minute flux, the public arena is changed every week or even day, and the legal arena is also moving fast with new legislation swiftly made. Furthermore, one could hypothesize that the typical sequence has changed. Instead of laws generally being changed after public opinion, authorities have established laws before or even contrary to public support. Authorities set normal laws aside, and start using rarely used or even obsolete laws.

A common sequence during COVID-19 has been that the prime minister has executed direct orders—for schools to shut down, people to shelter-in-place, curfews, and so on. Accordingly, the prime minister's order created an involuntary serviced office in your bedroom. Suddenly a number of organizations who never interact had to collaborate: the employers and schools of the household members sent their work tasks into the same bedroom. A need for resources emerged, for desks, Wi-Fi, and computers. Issues of ethics, health, and security unanswered, and which organization would be responsible for anything else than the demands on their workers. In theoretical terms: with great ambiguity concerning job demands and resources (Bakker and Demerouti, 2007), contracts psychological (Thompson and Hart, 2006) and legal alike, as well as norms for appropriate behavior (Kant and Norman, 2019).

However, societies seem to approach formulating, implementing and communicating their influence differently. The clarity and firmness vary, reminiscent of the norms of tight and loose cultures (cf. Gelfand et al., 2011). China and Norway implemented clear and strict rules early, e.g., with laws and police sanctions against staying at summer houses in Norway. Sweden implemented recommendations, which seemed more to play on internalized norms, lest one was to suffer shame and annoyed looks. The federal administration of the US, however, presented vague, inconsistent and contradictory statements, particularly from President Trump himself. Trump's statements were often in stark contrast to those of medical expertise and other officials (cf. Brennen et al., 2020; The Lancet, 2020). The gradual opening of societies has largely followed similar patterns. How well these strategies will work is a question for future research. We hypothesize that the looser strategies will increase variance in norms and behavior, and accordingly result in more norm conflicts, in particular as societies open up. Thinking ahead and creating predictability is part of elementary crisis management in order to proactively combat the main problems, and also part of minimizing the turmoil associated with re-negotiation of norms.

Societal changes in the public arena may also influence work tasks. Poor workers who have no choice to telecommute, may work under the gun to provide for their families (cf. CNN, 2020; Lind, 2020). They may be forced to work under conditions contrary to authority recommendations, or to view the latter as illegitimate (cf. Tyler, 2006) and end up at the state legislature of Michigan with guns.

Public events may lead to sudden changes for entire professions and their work tasks (cf. Abbott, 1988). An example concerns the debate on professional tasks for the US police force which surged following the killing of George Floyd by police, in the midst of the pandemic (Hill et al., 2020). Strong arguments were made to “defund the police”, in terms of stripping funds and professional tasks such as dealing with substance abuse, marital interventions, homelessness, and many other tasks from the heavily funded and heavily armored police.

#### The Level of Leadership/Organization: Unclear Boundaries Between “At Work” vs. “Off Duty”

In the COVID-19 situation, a vast number of (mostly) office workers have had to move their workplace to the home arena. For most, this has happened suddenly and without sufficient time to make optimal adjustments to technical equipment, furniture, etc. In addition, many have household members in a similar situation. For instance, one's partner might be working from home, and children in the household be home schooled or stay at home because childcare is closed.

In this situation, drawing the line between being “at work” vs. “off duty” may feel even more difficult than usual. One reason is reduced physical distance between the home and work arenas. Combined with various government recommendations/regulations that restrict people's opportunities for transport, leisure activities and social contact, this could result in feelings of constantly being “trapped” in a semi-work situation where it is difficult to declare to oneself and to others that “I'm off duty.”

##### Perceived Psychological Distance to the Organization

Related to this is the perceived distance between the individual and the organization. Chen and Li (2018) have specifically looked at employee-organization psychological distance, in an attempt to better address interpersonal psychological distance, which they argue is limited in CLT (Trope and Liberman, 2010). Chen and Li (2018) constructed and tested a self-report inventory to measure the various dimensions of employee–organization psychological distance. We here briefly outline each of these suggested dimensions. *Experiential distance* refers to how the individual perceives the future of the organization, based on how they assess a current experience or trend. *Behavioral distance* is the individual's perceptions about their affinity for the organization. *Emotional distance* refers to individual's emotional experience in corresponding and interacting with the organization. The authors exemplify this as the perceived “sense of oneness,” “sense of honor,” and “sense of experience.” *Cognitive distance* is the individual's perceived affinity for their organization, in terms of value orientation and personality consistency. *Spatial-temporal distance* refers to how close the individual feels that the organization is in space and time based on their level of involvement and understanding. Finally, *objective social distance* is the felt distance to the organization based on how closely one identifies with it.

##### COVID-19 Leading to an Increase in Emotional and Spatial-Temporal Distance to the Organization

Working from home during COVID-19 might in particular increase the employee's emotional and spatial-temporal distance to their organization. The organization's “affinity” for the worker may be reduced if the worker is less visible (cf. Elsbach et al., 2010). If the organization is at risk, it could also increase experiential distance. At the same time, other forms of employee-organization psychological distance could be reduced in the current situation. This applies in particular to cognitive distance, cf. the fact that any major crisis could force an organization to re-define and/or communicate more clearly around its core values.

As described earlier (The Level of Work Tasks: Norm Conflicts Relating to What Is the “True Essence” and Prioritized Tasks of One's Job), COVID-19 may influence distance to the work task. Especially for telecommuters, the work task may come extremely close, invading every aspect of private life. This could be even when the organization itself and its members would feel unusually distant. Norm violations include not only two parties, but a violating act such as a changed work task or job demand. Kant and Norman (2019) suggest that psychological distance needs to be considered between the two parties, the violating act, and the relative relationship between the three. Thus, we think that adding the work task and the relative distance between the task, the organization and the individual worker, may be an interesting amendment to the Chen and Li (2018) view of the relationship to the organization as an interpersonal relationship between two parties.

Overall, leaders should attend to decreasing psychological distance to job resources, and to increasing psychological distance to job demands (cf. Bakker and Demerouti, 2007). At least markedly protect against unnecessary invasion of private, off duty life. This may be hard for leaders in a crisis situation, where their knee-jerk reactions are likely to attempt increased control, and sending out signals whilst having limited capacity to respond.

#### The Interpersonal Level: The Influence of Your Work on Work/Private Life of Others, and the Influence of Others' Work on Your Work/Private Life

We now turn to the possible impact of unclear organizational boundaries and expectations upon the interpersonal level. How does work influence one's private life during COVID-19? And when several people are working from the same household, and several organizations are represented in the same home, how does one person's work influence the private life of others?

The consequence of working from home might differ between individuals. Kossek (2016) identifies 3 different styles that people adopt for dealing with work-life boundaries in a digital age. The styles represent different ways of dealing with 5 trends of modern work life: that work and non-work roles are increasingly blurred and overlapping (i.e., “boundarylessness”); that people are increasingly forced to work non-standard and specialized hours (“work-life customization”); the lack of control over when one is “on” vs. “off” work (“psychological control over working time”); an increase in interruptions between work and private life (“work—life fragmentation”); and the fact that people have multiple social identities (“diversity and inclusion”).

During COVID-19, people previously not working from home have now been forced to do so. This may impact various job demands and expectations about being “virtually present” at different hours than before. Thus, COVID-19 is likely to impact several of the 5 trends identified by Kossek (2016): in particular boundarylessness, work-life customization, psychological control over working time, and work-life fragmentation.

The 3 “work-life boundary management styles” identified by Kossek (2016) are integrator, separator, and cycler. An *integrator* is someone who has frequent work to non-work interruption behaviors and/or frequent non-work to work interruptions, either because this is their preference (“fusion lover”) or because they feel they have no other choice (“reactor”). A *separator* has a low frequency of both work-to-non-work and non-work-to-work interruptions, either by conscious choice (“divider”), or because the nature of the workplace prevents such interruptions (“captive”). A *cycler* is someone who periodically, be it weekly or seasonally, separates between work and non-work, and at other times integrates the two.

Based on these distinctions, one might predict that working from home would be less stressful for integrators or cyclers, but more stressful and difficult for separators. Moreover, the extent to which one's private life is influenced by other household members' work might depend on the other person's management style, as well as the degree of correspondence between one's own and the other person's work-life boundary management styles.

### Suggestion 3: Norm Conflicts Are Enhanced by Digitalization

So far, we have addressed and exemplified the types of norm re-negotiations taking place during COVID-19 (Suggestion 1), and how the stress associated with such re-negotiations may be enhanced due to the diffusion of the demarcation among various professional arenas and the private sphere (Suggestion 2). We now turn to the role of *digitalization*. In our view, the role of digital communication has the potential of enhancing norm conflicts and ambiguity. During COVID-19, digital communication is common when working from home. Obviously, the following arguments apply to the COVID-19 telecommuters. However, because digital communication has the same benefits and limitations regardless of geographical distance between different parties, the following arguments apply beyond the mitigation-efforts of COVID-19: to anyone communicating digitally, such as everyday telecommuters or members of a virtual team.

#### The Level of Work Tasks: The Impact of Digitalization on Norm Conflicts Related to Work Tasks

It is difficult to give general descriptions of how increased digitalization influences people's work tasks. This would depend on a broad variety of factors, including the nature of the work, the degree of active collaborations, and the degree of autonomy. Here we mainly discuss how increased digitalization could enhance norm conflicts related to work tasks.

When previously “off-line” tasks suddenly have to be conducted online/digitally, the workload of that task could change. Certain things become easier and less time-consuming with digitalization, others harder and more time-consuming. For instance, online meetings can sometimes be more efficient than face-to-face meetings. In contrast, follow-up on the work of individual pupils in a school class can be more time-consuming than the same follow-up in a classroom. At a more general level, digital teaching could benefit some children and disadvantage others.

Digitalization could also change interactions and division of labor in the workplace. Signals may be sent out, with people *ghosting* you, that is overlooking messages, posts and requests. Ghosting is far easier in the virtual context than face to face. Personal ability to explicitly ask or even demand help may not be enough to counter lack of resources in form of clear roles (cf. Xanthopoulou et al., 2009). However, with clear organizational support, such personal resources may mediate into efficient team work.

Certain professions face particular concerns during COVID-19. For example, counselors and therapists conducting confidential conversations with clients that now have to be conducted using online meeting platforms. Similarly, leaders having meetings about sensitive topics online. Although digital tools may have advantages also in these types of situations, e.g., because of efficiency or logistics, they also come with some risks and ethical concerns.

A fundamental challenge is how to best interact with and deal with larger groups. The one-on-one conversation may be quite easily replaced. Group processes, however, often need more preparation, a high grasp of technical demands and resources, and a well-adapted pedagogical process.

#### The Level of Leadership/Organization: The Impact of Digitalization on Leadership Distance

##### Automated Leadership

Digitalization in general is full of automated functions, defining tasks, times, sending reminders, insisting on follow-up, and so on. This is true also of digital resources in work-at-home conditions. We are accordingly convinced that automated functions vastly *increase interaction frequency*. Various gadgets can go “ping” in your pocket at virtually any time. The *physical distance is accordingly vastly reduced* for the automated leadership. The automated boss becomes more present, perhaps even looming, and unrelentingly so (cf Dzieza, 2020). It is however an open question whether the automated boss can be considered socially close (cf Antonakis and Atwater, 2002). Our suspicion is that the inhuman faceless quality of most *automated systems is more akin to an extremely socially distant lord*, than an empathic team leader. The automated leadership will potentially make greater relative impact amongst white collar workers during the COVID-19 changes, in a fashion previously experienced mainly by blue collar workers (cf. Dzieza, 2020). There are clear parallels to the scientific management/Taylorism in the early 1900-hundreds. The image of the conveyor belt literally swallowing the Tramp in Chaplin's “Modern Times” springs to mind. In light of the COVID-19 crisis, we note that such mechanistic organizational models are “designed to achieve predetermined goals … have difficulty in adapting to change … [and] are *not* designed for innovation (Morgan, 1998, p. 32).”

##### Human Leader Through Digital Interface

The human leader is likely to be more physically distant in the work-at-home condition. Here the digital interface may reduce this distance, for better and for worse. For the better through possible personal contact. For worse, by unprecedented invasion of the private sphere. Interaction frequency may both increase and decrease. Many interactions could be experienced as part of the automated leadership. The digitalization influence on social distance is perhaps the most difficult to predict in terms of directionality. Digitalization may likely act as a catalyst. That is, dependent on how leaders, formal or informal, use the digital resources. What the subordinate needs and expects is likely important to begin with. The leader could use the digital resources to increase or decrease social distance. Dependent on whether this does match or mismatch the needs of the subordinate, it could lead to either a desirable or an undesirable change in social distance. Accordingly, the leader may be experienced as both active or passive, as both constructive or destructive in this regard. If the subordinate acts with mismatching OCB or with CWB, the leader may through the digital interface be less able to appropriately and constructively remedy the situation.

#### The Interpersonal Level: Proper vs. Improper Behavior in Online Work Environments

##### Empirical Value of Clear Norms in Online Work Environments

Gajendran et al. (2015) found that telecommuting in general lead to higher perceived autonomy and was positively related to various outcome measures. This study was conducted some years ago, and in a setting where telecommuting was less common. For the purpose of our paper, the most important finding is that normative appropriateness of telecommuting was a central mediating variable. An important implication for the COVID-19 situation is that the norms of telecommuting matter. With the overarching changes of norms we argue in the wake of COVID-19, also veteran telecommuters may experience drops in performance and autonomy. It may for instance be more motivating to feel special, almost unique, in using these resources. When everybody does it, then there is a need to find other ways to increase workers' felt autonomy and motivation.

##### Matching the (Machine) Management Expectations

Elsbach et al. (2010) showed how *passive face time* has increased the view of subordinates as good employees, eligible for pay raises and promotions. It is not clear whether this also happens during and after the COVID-19 distance work. Is it still as proper for coworkers to hang around digitally and just showing their faces? Or will actual results weigh more? Face time as a proxy for organizational commitment may increase gender inequality (Stamarski and Son Hing, 2015), and would matter for caretakers of children. Now, strategies for appropriate subordinate appearances, may even with telecommuting include literal passive face time. An example is attending video meetings without contributing substantially. Yet, coworkers may struggle for new alternatives, such as inserting empty meetings into the digital calendar. All such struggles should be taxing. Initial performance increases may simply be reflective of initial absence of skills in digitally displaying passive face time. Furthermore, should subordinates fail to figure out new strategies, they may well experience the Tayloristic effects described by Dzieza (2020): “To satisfy the machine, workers felt they were forced to become machines themselves.” Thus, worker strategies in these new telecommuting conditions may indeed be interpreted as inefficient shirking, but also as strategies rewarded with career advantages (cf. Elsbach et al., 2010), and strategies to preserve humanity and health (cf. Dzieza), and to preserve or gain professional control of work tasks (cf. Abbott, 1988). It is also conceivable that subordinate behavior seemingly at odds with the machine or management definition of efficiency may be expressions of valuable emerging *shared mental models* of crisis solutions: Carrington et al. (2019) reported empirical findings that shared mental models during prolonged crises tended to emerge bottom-up, rather than top-down from management.

We have thus outlined a number of functional explanations of subordinate behavior which may fail to match the expectations of the automated or human management systems. Curiosity and cautious interpretation among managers and researchers seem called for.

##### Matching Interpersonal Expectations

Interpersonal proper behavior may be more ambiguous for most people new to the virtual environment (cf. Arvedsen and Hassert, 2020). Many cues are different than in real life: When to speak, when not to, when to raise your voice in affect, when not to. Particularly cues for “reading the room,” including pheromones or peripheral vision or small sounds may be heavily reduced or even absent. When relative power may be changed, for instance by new formal or informal leaders emerging, and old leaders receding into the background the potential for mismatched sweet spots will likely increase (cf. Kant and Norman, 2019).

### Examples of Specific Testable Propositions Derived From Our Suggestions

It is now time to give a few examples of specific propositions that can be derived from the above suggestions in combination. The suggestions in turn rest upon the selected four sets of theory.

Our aim is not to give an overview of every proposition possible to derive from combining our suggestions. Instead, we will exemplify two possible propositions, each relating to some of the suggestions outlined in The COVID-19 Situation Leads to a Massive Re-negotiation of Norms Related to Work, The COVID-19 Situation Diffuses the Demarcation Between the Various Professional Arenas and the Private Sphere, and This Diffusion Enhances the Stress Associated With Norm Conflicts, Norm Conflicts Are Enhanced by Digitalization.

The following propositions aim to show the practical applicability of our arguments. Researchers may be able to generate many other propositions based on the rationales of our suggestions. Propositions could be used as a basis for testable hypotheses and study designs. In addition, they could be used by leaders and other practitioners to inform day-to-day decisions, when they find themselves in notable changes, such as the ongoing pandemic. We attempted propositions that specify under which conditions certain of our theoretical suggestions are likely to emerge (Sutton and Staw, 1995).

Before testing any of the propositions, one needs to be aware of the importance of timing, as also highlighted by Sutton and Staw (1995). For both researchers and practitioners, the timelines presented in Figure 2 can be used to inform decisions on which questions to ask and which outcomes to expect, at different points in time. It can be used to indicate at which stage the crisis may have a confounding influence on other measures. For instance, in high intensity phases, through a higher likelihood of emotional responses such as irritation, anxiety, and fear; through lower psychological safety in their team at work (Edmondson, 1999); through higher role ambiguity and role conflict; or through lower identification with the organization.

#### Proposition 1

*Automated leadership is more likely to be experienced as negative during intense norm re-negotiation phases (cf. Norm Conflicts Are Enhanced by Digitalization, cf*. *Figure 2**). Therefore, each single leadership action will have an increased risk of being experienced as malign*.

By automated functions we do not mean technology itself, but functions in the organization, in software and other digital systems which may be experienced as leadership functions (cf. The Level of Leadership/Organization: The Impact of Digitalization on Leadership Distance). Rather than being perceived as job resources or tools for workers controlling work tasks such exposure to automated leadership will be experienced either as reduced job resources or increased job demands.

Rationale for this proposition can be found in our theorizing above. The likelihood of automated leadership being experienced as negative will increase with: intensity of renegotiation of norms in certain phases (Figure 2); signals invading the private sphere of the worker, or the private sphere of innocent third party household members (cf. The Interpersonal Level: The Influence of Your Work on Work/Private Life of Others, and the Influence of Others' Work on Your Work/Private Life); the close distance effect of high frequency of interaction (cf. The Level of Leadership/Organization: The Impact of Digitalization on Leadership Distance); signals carrying little face value of importance to the worker (cf. The Level of Work Tasks: Norm Conflicts Relating to What Is the “True Essence” and Prioritized Tasks of One's Job); reducing the workers' control of work tasks [cf. The Level of Work tasks: The Continuous Influence of Societal Changes on Work Conditions and Work Tasks (and Private Tasks)] or job resources in comparison with job demands (cf. The Level of Leadership/Organization: Norm Conflicts Relating to Who or What Has Oversight, Surveillance, and Sanctioning Rights).

Leaders or practitioners considering the proposition in an ongoing crisis, may just like researchers want to keep track of and measure the phenomenon. Considering their responsibility of caring for both the survival of their organization and their employees, they may want to:

(a) Reduce automated leadership to a minimum, particularly functions with limited proven benefit to the organization and high likelihood to encumber the workers. In this, they should take pains to protect the workers private sphere. (b) Strictly prioritize critical tasks, and to communicate clearly around these. Communication and discussion on the topic of what the current norms for desirable and undesirable behavior are should be done on a regular basis. (c) Show empathy and concern for the real threats workers likely discern on the horizon. This would reduce unnecessary resistance against organizational change (Oreg, 2006) by building trust, but above all display a uniquely warm and beneficial aspect of human leadership countering the detriments of automated leadership we hypothesize.

#### Proposition 2

*Organizations which take pains to proactively deal with the intense norm re-negotiation phases and associated issues will fare better than organizations which do not take such pains. Organizational outcomes may include, but not be limited to: organizational survival, increased profit or other results, increased market shares, increased reputation; increased innovation; lower turnover; increased trust in management; lower sick-leave; increased identification with the organization; increased psychological safety; increased team cohesion; increased leader–member exchange; increased role clarity; decreased role conflict; lower reports of destructive leadership; higher reports of constructive leadership*.

Rationale for this proposition can be found in our theorizing above. Ways in which organizations can deal with norm negotiations may include: timely efforts to parry both escalation and de-escalation phases (cf. The COVID-19 Situation Leads to a Massive Re-negotiation of Norms Related to Work; Figure 2); efforts aimed at acknowledging threats and stressors experienced by workers; efforts aimed at fair and explicit re-negotiation of norms (cf. The COVID-19 Situation Leads to a Massive Re-negotiation of Norms Related to Work); minimization of low priority stressors (cf. The Level of Work Tasks: Norm Conflicts Relating to What Is the “True Essence” and Prioritized Tasks of One's Job); minimization of non-critical automated leadership (cf The Level of Leadership/Organization: The Impact of Digitalization on Leadership Distance); maximization of low distance human leadership supporting workers (cf. The Level of Leadership/Organization: The Impact of Digitalization on Leadership Distance); minimization of signals invasive of the private sphere (cf. The Interpersonal Level: The Influence of Your Work on Work/Private Life of Others, and the Influence of Others' Work on Your Work/Private Life).

Leaders or practitioners considering the proposition in an ongoing crisis, may just like researchers want to keep track of and measure the phenomenon. Considering their responsibility of caring for both the survival of their organization and their employees, they may want to: use best practice in general crisis management (e.g., Lunde, 2014)—specifically to use mental time travel (Nyberg et al., 2010) anticipating the high intensity phases of norm re-negotiation. Then to let this guide preparatory focused and prioritized efforts in line with the previous paragraph. Above all—passivity is not an option!

## Concluding Remarks

The COVID-19 pandemic poses great uncertainty. There are uncertainties about the duration and extent of the pandemic, and of the preventive and mitigative measures. We have argued that the COVID-19 pandemic and its associated crisis trajectories represent a context of massive re-negotiation of norms in a number of arenas. Due to the uncertain nature of COVID-19, the re-negotiation of social norms may appear correspondingly fleeting. People will wonder how long they will have to be working from home, whether the new norms will be carried into the future, and/or whether these will dissolve when life returns to normal. We have discussed possible implications of this impermanence.

This paper provides explanations of ongoing, urgent issues causing substantial changes in the norms of working life. This allows for the general predictions we have provided in our three main suggestions, with subsequent more specific suggestions. Using these will allow researchers to easily build yet more specific propositions, which we have given two examples of. Further theoretical development and empirical testing may be done on this foundation.

The individual theories from which our suggestions are derived, are well-established and empirically supported. Our contribution is to clarify where the selected theories have connections, and how they together may hold even greater explanatory value than alone. We expect the phenomena we describe to have distinct effects. Our suggestions connect to well-documented general advantages, for instance creating role clarity, psychological safety, and resolving jurisdictional contests. Therefore, they could be applied by leaders who are facing norm negotiations during the intensity-changes of this pandemic or other significant crises (cf. Figure 2).

Our analysis is theoretical. Our suggestions and discussion points are derived from applying and combining ideas from 4 different theoretical approaches. Our hope is that our theoretical analysis can be used as a framework for understanding how people's work conditions are affected by COVID-19. The suggestions derived from our theoretical analysis can also be used as a starting point for empirical studies. A limitation related to the choice of a theoretical genre, is that we could have applied other theories, provided other examples and looked at other levels of analysis.

A number of factors may modify the renegotiation of social norms, many of which have not been discussed here. These include *culture* (e.g., tight and loose; Gelfand et al., 2011), social distance (Kant and Norman, 2019), and leadership distance (Antonakis and Atwater, 2002). In the current situation, the most obvious modifying factor is *digitalization*. With social distancing measures, work-related communication largely occurs digitally. The situation also opens for leaders controlling/surveilling their workers by digital means. We therefore also address some concerns arising from the fact that for most telecommuters, relevant interactions relating to social norm re-negotiation during COVID-19 take place *in digital worlds* and *simultaneously* in people's homes. We suggest that re-negotiation of norms in the COVID-19 situation may be influenced by norm conflicts caused or enhanced by so-called “disrespectful technologies” (Diefenbach and Ullrich, 2019).

Even though this paper specifically addresses stressors for people currently employed, it is important to acknowledge the stress, uncertainty, and economical difficulties that now face those unemployed as a result of COVID-19. Our investigation centers around those who relatively speaking are fortunate during the COVID-19 pandemic. The challenges we address are about processes demanding change, adjustment and renegotiation; in confined spaces; where the demands of many organizational bodies meet. In the paper, we have gone into depth about each of our theoretical suggestions, provided illustrative examples and provided interpretation of relevant data. Our aim is to contribute to existing theory, including theory on negotiation of norms, professions, digitalization, and benign violations. Because we point to potential risk factors of psychological distress, our analysis also has long term applied value for clinical and organizational psychology. It is our hope that our analysis also is of value, as nearly half of the world in June 2020 craves to understand how to deal with the dilemmas we have described.

## Author Contributions

LK conceived of the presented idea and had the main responsibility for developing the theoretical arguments in line with existing literature. LK also had the main responsibility for writing up the paper. EN provided input into the organization of arguments, took part in discussions, and contributed to the write-up. Both authors contributed to the article and approved the submitted version.

## Conflict of Interest

The authors declare that the research was conducted in the absence of any commercial or financial relationships that could be construed as a potential conflict of interest.

## Publisher's Note

All claims expressed in this article are solely those of the authors and do not necessarily represent those of their affiliated organizations, or those of the publisher, the editors and the reviewers. Any product that may be evaluated in this article, or claim that may be made by its manufacturer, is not guaranteed or endorsed by the publisher.
